# Agency in schizophrenia and autism: a systematic review

**DOI:** 10.3389/fpsyg.2023.1280622

**Published:** 2023-12-21

**Authors:** Denise P. W. Tan, Olivia Carter, Darcy-Rose Marshall, Kelsey Perrykkad

**Affiliations:** ^1^Melbourne School of Psychological Sciences, The University of Melbourne, Melbourne, VIC, Australia; ^2^Monash Centre for Consciousness and Contemplative Studies, School of Philosophical, Historical and International Studies, Monash University, Melbourne, VIC, Australia; ^3^Centre for Women’s and Children’s Mental Health, Department of Psychiatry, School of Clinical Sciences, Faculty of Medicine, Nursing and Health Sciences, Monash University, Melbourne, VIC, Australia

**Keywords:** sense of agency, judgment of agency, transdiagnostic, schizophrenia, autism

## Abstract

**Introduction:**

Previous research suggests that altered experiences of agency are an underlying vulnerability in both schizophrenia and autism. Here, we explore agency as a potential transdiagnostic factor by conducting a systematic review of existing literature investigating agency in autism and schizophrenia individually and together.

**Methods:**

Following the Preferred Reporting Items for Systematic Reviews and Meta-Analyses (PRISMA) guidelines, we conducted three systematic searches on PsycINFO, Embase, Medline, PubMed and Web of Science to identify studies that investigated (1) agency in schizophrenia, (2) agency in autism, and (3) agency in both schizophrenia and autism.

**Results:**

A total of 31 articles met eligibility criteria for inclusion and data extraction, with 24 measuring agency in schizophrenia, 7 investigating agency in autism, and no articles comparing the two. Results show that, compared to control populations, agency is significantly different in every identified schizophrenia study and generally not significantly different in autism.

**Discussion:**

Importantly, we identified a lack of studies using common tasks and a disproportionate number of studies investigating different dimensions of agency across the two conditions, resulting in limited grounds for valid comparison.

**Systematic review registration:**

Prospero, CRD42021273373.

## 1 Introduction

Experiences of agency include concurrent feelings of control over one’s actions and their sensory consequences (sense of agency) and a retrospective report of “I did that” (judgment of agency) (Synofzik et al., 2008; Moore, 2016). Altered experiences of agency are associated with psychiatric conditions and often result in significant functional impairments and distress (Balconi, 2010; Hur et al., 2014). Agency has been identified as a key transdiagnostic construct under the National Institute of Mental Health’s Research Domain Criteria (RDoC).

Schizophrenia and autism are two (of several) psychological conditions that are thought to be associated with altered experiences of agency (Hur et al., 2014; Sperduti et al., 2014; Zalla and Sperduti, 2015). However, it is unclear to what extent these alterations represent changes to common processes or distinct differences in each condition. Some recent studies have explicitly claimed that these two conditions are diametrically opposed in sense of self including embodiment and agency (Crespi and Dinsdale, 2019; Benítez-Burraco et al., 2021). Further, autism and schizophrenia have a shared history, with autism first being defined as a part of schizophrenia, as the symptom of being withdrawn in the DSM-I and DSM-II, and only becoming its own condition in the DSM-III (American Psychiatric Association, 1952, 1968, 1981). As such, while agency is proposed as a transdiagnostic factor which may be used to distinguish a range of psychiatric conditions, comparing the evidence for agency differences across these two conditions is an important step in developing a broader multidimensional transdiagnostic approach.

If autism and schizophrenia represent polar ends of an agency spectrum, this may serve as a diagnostic boundary and inform treatment decisions in clinical practice. Should the agency alterations be similar instead, altered agency may be a factor shared between schizophrenia and autism. This may inform future research into the underlying mechanisms of these conditions. However, the strength of the evidence supporting these alternatives is yet to be assessed.

Furthermore, it is unknown whether both literatures have addressed the same experiences of agency. Research investigating sense of agency tends to employ implicit measures while research investigating judgment of agency tend to rely on explicit measures (Moore, 2016). For example, the intentional binding paradigm (Haggard et al., 2003) is an implicit measure that uses the shortening of the perceived time interval between a self-generated action and its consequence to indirectly estimate one’s sense of agency. A recent transdiagnostic review of intentional binding showed differences in intentional binding in schizophrenia and its spectrum, but inconsistent findings in autism (Moccia et al., 2023). Meanwhile, explicit measures of agency can be used in conjunction, requiring the participant to directly self-report their agency in words, or using a Likert scale (Moore, 2016). Siebertz and Jansen (2022) found that two distinct intentional binding paradigms generated divergent outcomes in terms of sense of agency. This highlights a critical need to not only distinguish which agentic experience is being investigated, but also the type of measures used in each body of research.

To date, no studies have systematically reviewed the empirical literature across experimental paradigms for evidence regarding different factors that contribute to experiences of agency within these conditions or transdiagnostically for any set of conditions. Notably, a preliminary screen suggests a comparatively low number of autism studies, as such, a scoping review is warranted to provide an assessment of the current state of the literature to inform further research. Hence, this study aims to determine whether individuals with schizophrenia and autism demonstrate altered agency, examine the availability of evidence comparing agency in autism and schizophrenia and whether the independent literatures provide comparable evidence to inform transdiagnostic goals.

## 2 Methods

This scoping review was conducted in accordance with the Preferred Reporting Items for Systematic Reviews and Meta-Analyses (PRISMA) guidelines (PRISMA-P Group et al., 2015). The methods were documented in a protocol that was registered and published on the PROSPERO database (CRD42021273373). No informed consent was required.

**Figure 1:**
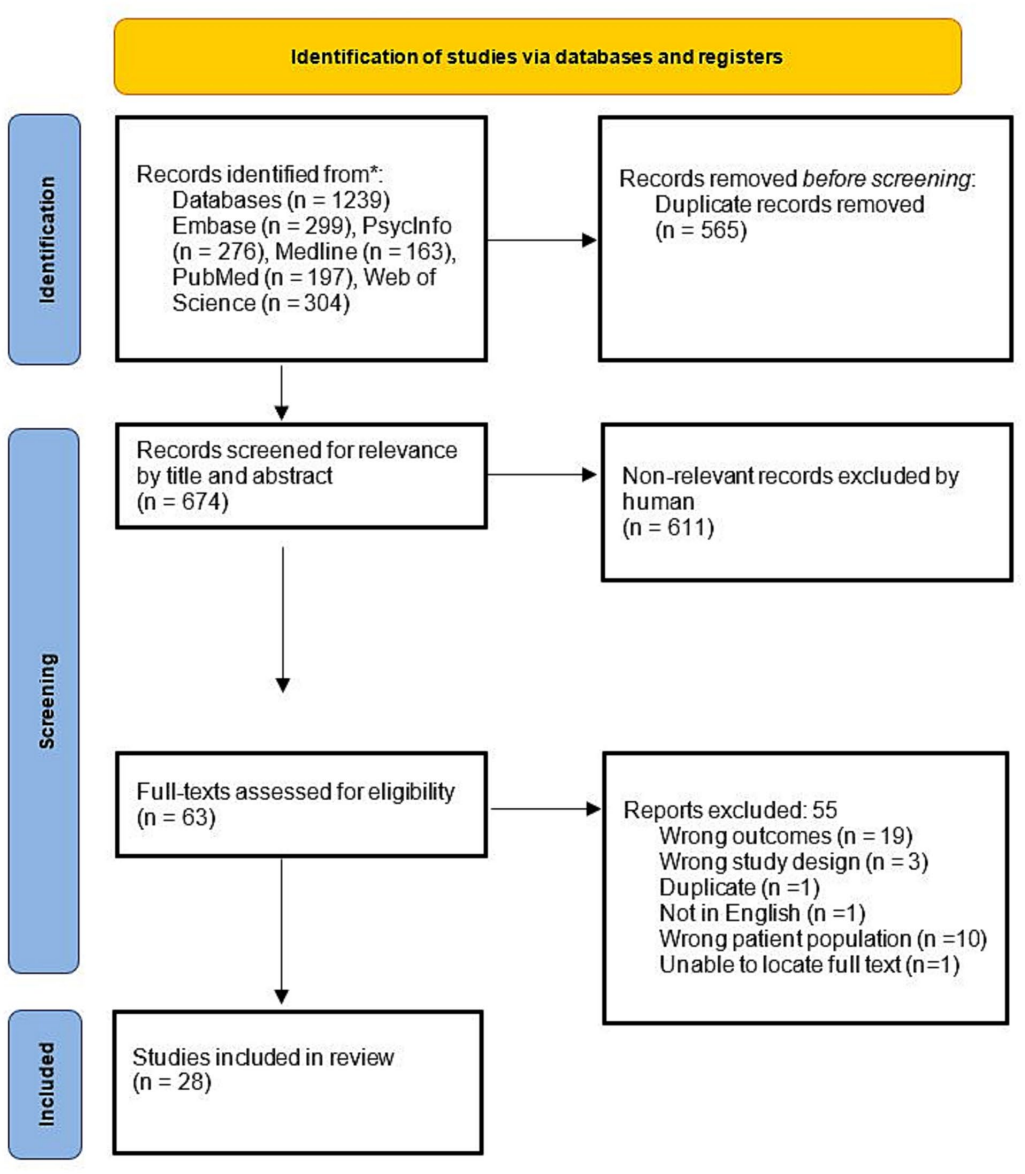
PRISMA flow diagram representing the selection of studies included in review of agency in schizophrenia, autism and both populations (as at 26 July 2021).

**Figure 2:**
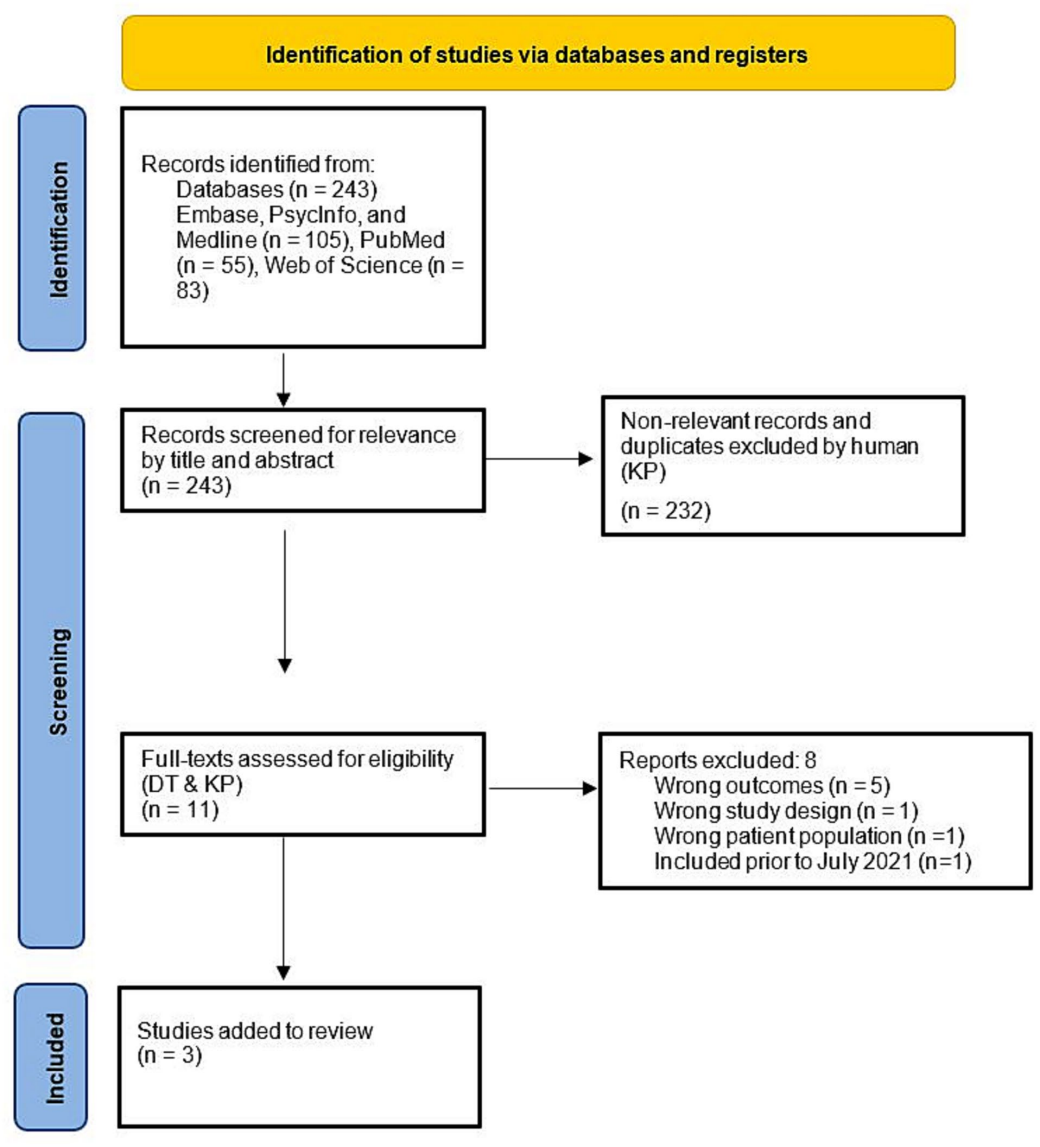
PRISMA flow diagram summarizing update of studies included in review of agency in schizophrenia, autism and both populations between 2021 and 8 May 2023.

The searches were conducted across the electronic databases PsycINFO, Embase, Medline, PubMed and Web of Science, and included only studies written in English in peer-reviewed journals. The first database search was conducted on 26 July 2021, and was repeated on 8 May 2023, indicating three new articles for review. Three separate searches were conducted to identify studies that investigated (1) agency in schizophrenia, (2) agency in autism, and (3) agency in both autism and schizophrenia (see Supplementary material for search terms and screening criteria). In brief, to be included, studies needed to include human participants in a diagnosed clinical group and include a neurotypical comparison group in a case-controlled design, outcomes were required to assess the participant’s own judgment/sense/feeling of agency behaviorally (implicitly or explicitly but not solely using biological proxies). See Supplementary material for search strategy details and complete eligibility and selection criteria. The screening process, comprising stages (1) pilot (2) title and abstract screening, and (3) full-text screening, for eligibility was performed independently by DT and DM using Covidence, with conflicts resolved by KP. The resultant papers yielded from screening were then appraised (DT and DM) for the internal and external validity of outcomes using a quality appraisal checklist (The National Institute for Health and Clinical Excellence, 2012). See Figures 1, 2 for the PRISMA flow diagrams.

## 3 Results

**Figure 3:**
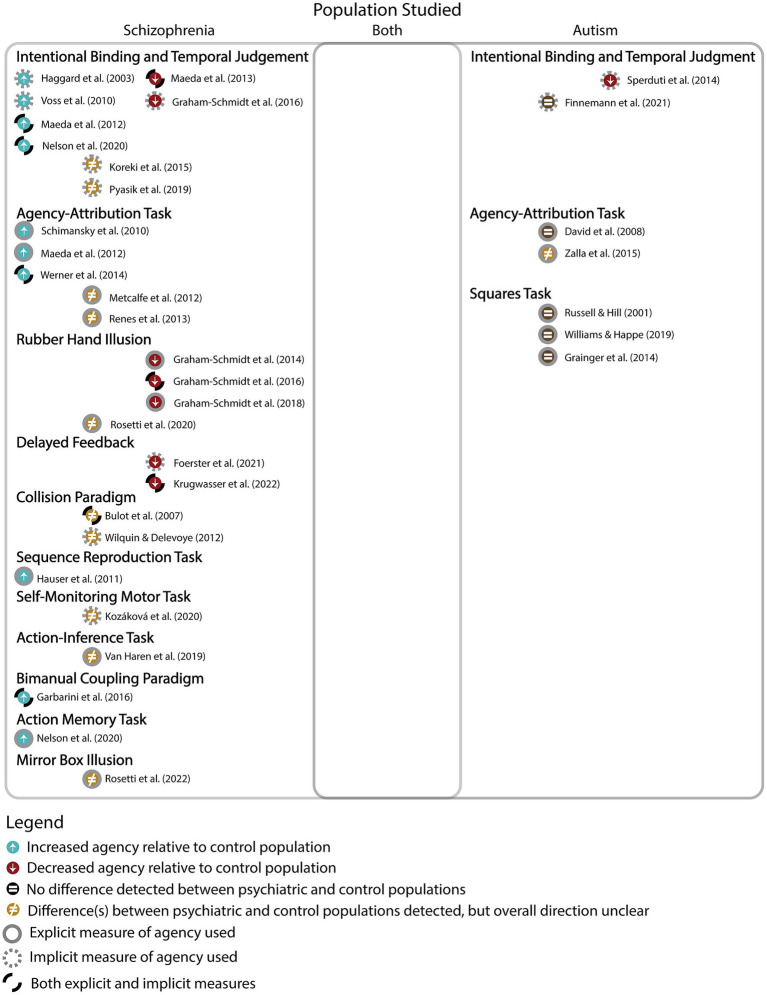
This figure provides an overview of the results indicating a disproportionately greater number of agency studies in schizophrenia than autism, with no studies investigating agency in both conditions. The results are further categorized into type of task, and whether they were implicit or explicit measures. The figure also reveals results of individual studies investigating whether agency was increased, decreased, different, or not different when comparing clinical and control groups.

A total of 28 of 1,239 retrieved articles met eligibility criteria for inclusion and extraction of data at the initial search (July 2021), and 3 were added when the search was updated in May of 2023, for a total of 31 eligible articles. Of these articles, 24 investigated agency in schizophrenia, 7 investigated agency in autism, and no articles directly compared the two. A wide range of tasks were employed to measure agency with little overlap in types of measures used across conditions (Figure 3). The most common task used in schizophrenia was the intentional binding task or similar implicit variants of temporal estimation tasks (implicit) and in autism it was the squares task (explicit). The intentional binding task was the sole common task across groups. It should be noted that while classically the rubber hand illusion measures sense of ownership, which is distinct from sense of agency, the rubber hand illusion studies identified were included as part of this study because they included an explicit or implicit measure of agency alongside the classic ownership paradigm. See complete data extracted from reviewed articles in Table 1 for the Schizophrenia search and Table 2 for the Autism search.

**Table 1:** Summary of findings from primary outcome measures and participant characteristics relevant to agency in Schizophrenia.

Study	Country	SSC (N), Gender (% male)	Age (SD), range	Medica-tion (Y/N)	Diagnosis (Diagnostic System)	SSC Sx Measure	Control (N), Gender (% male)	Age (SD), range	Agency Task	Type of Measure		Agency (Different/ Not Different/ Increased/ Reduced)
										Implicit/ Explicit	SoA/ JoA	
Haggard et al. (2003)	United Kingdom	8 75%	44.6 ±9.9	Y	SZ (DSM-IV)	SAPS, SANS	8 75%	42.3 ± 9.3	Intentional Binding	Implicit	SoA	Increased
Bulot et al. (2007)	France	24 NR	NR	Y	SZ (DSM-IV)	PANSS	24 NR	NR	Collision Paradigm	Implicit Explicit	SoA JoA	Different Different
Schimansky et al. (2010)	Switzerland	40 70%	38.0 ± 9.8	Y	SZ (ICD-10)	PANSS	40 57.5%	34.3 ± 9.7	Agency-Attribution Task**	Explicit	SoA	Increased
Voss et al. (2010)	Germany	24 92%	NR	Y	Paranoid-SZ (DSM-IV, ICD-10)	PANSS	24 91.6%	20.0–66.0	Intentional Binding	Implicit	SoA	Increased
Hauser et al. (2011)	United States	60 72%	32.9 (9.9)	N	SZ (DSM-IV)	PANSS	30 50%	34.3 (11.3)	Sequence Reproduction Task	Explicit	JoA	Increased
Maeda et al. (2012)	Japan	30 70%	37.9 (11.9)	Y	Paranoid-SZ (DSM-IV)	PANSS	30 53%	35.9 (10.4)	Intentional Binding Agency Attribution Task**	Implicit Explicit	SoA JoA	Increased Increased
Metcalfe et al. (2012)	United States	22 41%	42.3 (11.1)	Y	SZ/Schizoaffective (DSM-IV)	BPRS, SANS	NR 45%	38.1 (11.3)	Agency-Attribution Task**	Explicit	JoA	Different
Wilquin and Delevoye-Turrell (2012)	()France	32 50%	13–23	Y	FEP, UHR (DSM-IV)	PANSS	36 36%	13.5–23.4	Collision Paradigm	Implicit	SoA	Different
Maeda et al. (2013)	Japan	50 64%	*	N	NS-SZ, Paranoid-SZ (DSM-IV)	PANSS	35 46%	35.0 (10.0)	Intentional Binding	Implicit Explicit	SoA JoA	Reduced (NS- predominant) Increased SoA & JoA (Paranoid-type)
Renes et al. (2013)	Netherlands	23 87%	28.5 ±8.6	Y	SZ (DSM-IV)	PANSS	23 82%	32.7 ± 7.1	Agency-Attribution Task**	Explicit	JoA	Different
Graham-Schmidt et al. (2014)	Aus	53 68%	*	Y	SZ (DSM-IV, ICD-10)	SAPS, SANS, PSI	48 50%	46.2 ± 1.7	Rubber Hand Illusion	Explicit	JoA	Reduced
Werner et al. (2014)	Germany	20 75%	37.1 (7.8)	N	Paranoid-SZ (DSM-IV-TR, ICD-10)	SAPS, SANS	18 72%	36.7 (8.9)	Agency-Attribution Task**	Implicit Explicit	SoA JoA	Increased Increased
Koreki et al. (2015)	Japan	30 57%	42.5 (9.4)	Y	SZ (DSM-IV-TR)	PANSS	30 43%	39.8 (11.2)	Intentional Binding	Implicit	SoA	Different
Garbarini et al. (2016)	Italy	20 50%	46.7 (14.7)	Y	SZ (DSM-IV-TR)	SAPS, SANS	20 50%	45.2 (12.4)	Bimanual Coupling Paradigm	Implicit Explicit	SoA JoA	Increased Increased
Graham-Schmidt et al. (2016)	Aus	39 67%	*	Y	SZ (DSM-IV, ICD-10)	SAPS, SANS, PSI	43 53.4%	44.6 ± 1.7	Intentional Binding Rubber Hand Illusion	Implicit Explicit	SoA JoA	Reduced Reduced
Graham-Schmidt et al. (2018)	Aus	51 71%	*	Y	SZ (DSM-IV, ICD-10)	SAPS, SANS, PSI	49 48.9%	45.9 ± 1.7	Rubber Hand Illusion	Explicit	JoA	Reduced
Pyasik et al. (2019)	NR	20 0%	20-63	N	SZ (NR)	NR	20 0%	23–57	Intentional Binding	Implicit	SoA	Different
Van Haren et al. (2019)	Netherlands	36 NR	NR	N	SZ (NR)	NR	36 NR	NR	Action-Inference Task	Explicit	SoA	Different
Kozáková et al. (2020)	Czech Republic	161 56%	*	Y	FEP (ICD-10)	PANSS	154 49%	*	Self-Monitoring Motor Task	Implicit	SoA	Different
Nelson et al. (2020)	Aus	89 45%	*	Y	FEP, UHR (DSM-IV)	BPRS, SANS	34 29%	21.09 (1.9)	Action Memory Task	Explicit	JoA	Increased Increased
									Intentional Binding	Implicit	SoA	
Rossetti et al. (2020)	Italy	31 58%	41.3 (14.1)	Y	SZ (DSM-IV-TR)	SAPS, SANS	36 18%	25.8 (7.9)	Rubber Hand Illusion	Explicit	JoA	Different
Rossetti et al. (2022)	Milan	29 73%	44.3 ±12.2	Y	SZ (DSM-IV-TR)	SAPS, SANS	32 38%	26.7 ± 9.8	Mirror Box Illusion	Explicit	SoA	Different
Foerster et al. (2021)	France	23 68%	37.9 ±2	Y	SZ	PANSS	22 45%	38.8 ± 1.9	Haptic Pointing Task – Delayed Feedback	Implicit	SoA	Reduced
Krugwasser et al. (2022)	Israel	30 100%	30.9 ±8.3	Y	SZ/ Schizoaffective/ Paranoid SZ/ Psychosis	PANSS	30 50%	24.4 ± 3	Embodied VR Paradigm – Delayed Feedback	Implicit/ Explicit	SoA	Reduced

The term “different” was used to connote “different from healthy control”.

*Overall mean age for people with schizophrenia not provided but mean age for specific clinical groups (e.g., First-Episode Psychosis, Ultra-High Risk, Negative Symptom-Predominant, or Paranoid-Type).

**Agency-attribution task used as a general label referring to agency tasks that involved a behavioral component asking about participants’ subjective rating of agency they felt over a self-initiated action and its consequences.

NR, not reported; SZ, Schizophrenia; NS, Negative symptom predominant schizophrenia; FEP, First Episode Psychosis; UHR, Ultra High Risk; DSM, Diagnostic and Statistical Manual of Mental Disorders; ICD, International Classification of Diseases; SANS, Scale for the Assessment of Negative Symptoms; SAPS, Scale for the Assessment of Positive Symptoms; PANSS, Positive and Negative Syndrome Scale; BRPS, Brief Psychiatric Rating Scale; PSI, Passivity Symptoms Interview; SIPS, Structured Interview for Assessment of Prodromal Symptoms; SOPS, Scale of Prodromal Symptoms; SPI, Schizophrenia Proneness Instrument.

**Table 2:** Summary of findings from primary outcome measures and participant characteristics relevant to agency in Autism.

Study	Country	ASD (N), Gender (% male)	Age (SD), range	Diagnosis (Diagnostic System)	ASD Sx Measure	Control/ Neurotypical (N), Gender (% male)	Age (SD), range	Agency Task	Type of Measure		Agency (Different/ Not Different/ Increased/ Reduced)
									Implicit/ Explicit	SoA/ JoA	
Russell and Hill (2001)	United Kingdom	28 75%	NR	AD, AS (DSM-IV-TR)	–	5	NR	Squares Task	Explicit	JoA	Not Different
David et al. (2008)	Germany	24 58%	32.3 ± 10.0	HFA, AS (ICD-10)	AQ	24	30.6 ± 5.1	Agency-Attribution Task**	Explicit	JoA	Not Different
Williams and Happé (2009)	United Kingdom	16 NR	NR	AD, AS, PDD-NOS (DSM-IV-TR)	–	16 NR	NR	Squares Task	Explicit	JoA	Not Different
Grainger et al. (2014)	United Kingdom	17 NR	29.1	AD, AS (DSM-IV-TR)	AQ	17 NR	29.4	Squares Task	Explicit	JoA	Not Different
Sperduti et al. (2014)	France	15 NR	33.5 ± 11.0	ASD (DSM-IV-TR)	AQ	17 NR	33.1 ± 11.1	Intentional Binding	Implicit	SoA	Reduced
Zalla et al. (2015)	France	19 84%	28.8(7.1), 20–45	HFA, AS (DSM-IV-TR)	AQ	19 89%	26.4 (6.1), 20–43	Agency-Attribution Task**	Explicit	JoA	Different
Finnemann et al. (2021)	United Kingdom	27 48%	NR	ASC (NR)	AQ	26 39%	NR	Sensory Attenuation, Intentional Binding	Implicit	SoA	Not Different

The term “different” was used to connote “different from neurotypical control”.

**Agency-attribution task used as a general label referring to agency tasks that involved a behavioral component asking about participants’ subjective rating of agency they felt over a self-initiated action and its consequences.

NR, not reported; DSM, Diagnostic and Statistical Manual of Mental Disorders; ICD, International Classification of Diseases; AD, Autistic Disorder (DSM-IV); AS, Asperger’s Syndrome (DSM-IV); HFA, High-Functioning Autism (DSM-IV); PDD-NOS, Pervasive Developmental Disorder Not Specified Otherwise (DSM-IV); ASD, Autism Spectrum Disorder (DSM-IV); ASC, Autism Spectrum Conditions; AQ, Autism Spectrum Quotient.

Overall, none of the studies comparing schizophrenia and controls found statistically indistinguishable performance on any agency task, with the most common results (10 of 24) indicating increased agency in participants with schizophrenia (i.e., over-attributed to themselves) relative to controls. A closer inspection suggested that such over-attribution may be associated with a subset of positive symptoms where participants with schizophrenia overestimate the causal reach of their actions in the external world (Voss et al., 2010; Maeda et al., 2012, 2013; Hur et al., 2014; Werner et al., 2014; *cf.* Foerster et al., 2021). Negative symptom presentations (Maeda et al., 2013) and passivity symptoms (i.e., delusions of alien control) were generally associated with reduced attribution of agency, as seen in 6 out of 24 articles (Graham-Schmidt et al., 2014, 2016, 2018). Additionally, the rubber hand illusion and related delayed feedback paradigms consistently show reduced agency in schizophrenic groups compared to controls (Graham-Schmidt et al., 2014, 2016, 2018; Foerster et al., 2021; Krugwasser et al., 2022; *cf.* Rossetti et al., 2020).

In studies investigating sense of agency in diagnosed autistic individuals, two of the seven studies showed altered agency (Zalla et al., 2015), with one of these specifically finding reduced agency in autism (Sperduti et al., 2014). The five remaining studies suggest no difference in agency between autistic and control participants.

No study directly compared schizophrenia and autism on measures of agency.

## 4 Discussion

This scoping review revealed that no study has directly compared agency between schizophrenia and autism. The types of measures used in schizophrenia are heterogenous, using a broad range of implicit and explicit measures of both sense and judgment of agency, while the autism literature is predominated by explicit measures of judgment.

The results indicate that agency is altered in schizophrenia, with more studies finding reports of both increased and reduced agency relative to control participants. This apparent contradiction may reflect difference in symptomatology (positive vs. negative symptoms) or stage of illness progression. Evidence suggests that people exhibiting increased agency are more likely to experience positive symptoms like delusions of reference, paranoia/persecution, and control over external objects, where they overestimate the causality of their actions in the external world (Haggard et al., 2003; Schimansky et al., 2010; Garbarini et al., 2016, *cf.* Foerster et al., 2021). In support of this explanation, this review identified four studies that focused on patients with predominantly paranoid-type delusions and consistently reported excessive agency (Voss et al., 2010; Maeda et al., 2012, 2013; Werner et al., 2014).

Papers that reported reduced agency in schizophrenia suggested two candidate explanations for this pattern of results. On one hand, people exhibiting reduced agency may be more likely to experience a different subset of positive symptoms than those showing increased agency including delusions of being controlled and thought insertion, underestimating the causality of their actions to themselves rather than external stimuli (Fourneret et al., 2001). This was observed in three papers reporting reduced agency that investigated patients experiencing predominantly passivity symptoms, referring to delusions of alien control/ being controlled by others (Graham-Schmidt et al., 2014, 2016, 2018). On the other hand, our synthesis also showed that patients with predominantly negative symptoms generally have reduced agency. Notably, Maeda et al. (2013) was the first and only study to date that investigated agency in schizophrenia comparing patients with predominantly negative symptoms and paranoid-type symptoms.

A possible explanation for this mixed/inconsistent association between symptomatology and agentic experience is the relationship between stage of illness progression and severity of altered agency. Although the number of studies investigating this relationship are limited, three studies have suggested a unified story that agency alterations increase across stage of disorder from prodromal ultra-high risk (where negative symptoms and attenuated positive symptoms predominate the clinical presentation) to the acute phase of first-episode psychosis (where previously attenuated symptoms have crystallized into full-blown positive and negative psychotic symptoms) (Wilquin and Delevoye-Turrell, 2012; Kozáková et al., 2020; Nelson et al., 2020). The severity of altered agency appears related to the severity of positive symptoms reflected by stage of illness. A 1-year follow study by Kozáková et al. (2020) further suggests that the severity of agency alterations is state-dependent and can attenuate as patients enters the remissive phase of the illness, which is often characterized by reduced psychotic symptoms and improved functioning. As such, it appears that severity of altered agency is more related to the severity of positive symptoms. However, given the paucity of studies examining this relationship, it will be important for future research to consider the stage of illness, comparisons of symptomatology across stages and longitudinal follow-ups. Together with the above findings, the overall finding is that agency is generally over-attributed in schizophrenia and is generalized to patients with predominantly positive symptoms that may reflect stage of illness.

Only a small number of studies have examined agency in autism. Within these, 5 studies suggested that agency is not different, and only 2 suggested that agency is altered, with 1 indicating reduced agency in autism compared to neurotypical participants (Sperduti et al., 2014). This pattern of results stands in contrast to assumptions that agency is altered in autism (Sperduti et al., 2014; van Laarhoven et al., 2019), possibly because the full range of agentive processes have not been studied (Zalla and Sperduti, 2015; Perrykkad and Hohwy, 2020). Of note, Finnemann et al. (2021) focused on agency in autism, but also found higher schizotypal traits in an autistic group compared to neurotypical controls. This reinforces a further need to consider diagnostic and trait-based overlaps when using dimensional approaches to distinguish autism and schizophrenia.

Overall, these findings suggest that agency in autism and schizophrenia are neither diametrically opposed nor decidedly similar in alteration, in contrast to such claims in the literature (Crespi and Dinsdale, 2019; Benítez-Burraco et al., 2021). Differences in self-processing in schizophrenia but not in autism is consistent with recent findings showing a significantly stronger association between self-concept clarity and schizotypy than with autism traits (Perrykkad and Hohwy, 2022). Further, there is little ground for comparison in the existing literature as not only have no studies compared agency in autism and schizophrenia directly, but there is also little overlap in the measures used in the two conditions.

Possible limitations of the current review include missing any articles that did not appear in the search results using the selected terms at the time of the searches, but otherwise appeared to fit the inclusion and exclusion criteria, including Franck et al. (2005). The findings of this study, showing increased temporal binding for some conditions (though notably not the agentive condition) for a diagnosed schizophrenia group compared to control participants are consistent with findings of the studies included in this review (Franck et al., 2005). Another limitation is the focus on only behavioral results and the exclusion of findings based on neural data. While neural data is indeed important, especially for mechanism discovery, psychological disorders remain defined clinically by behavioral symptomatology and clinician-patient interactions, so we focused here on behavioral and self-report findings. In the future, when inevitably more research has been done to fill some of the gaps identified by this review, researchers should consider repeating a review like this for a wider range of agency-related disorders, such as functional movement disorders, and a broader range of levels of analysis (such as those described by the RDoC matrix including for instance neurotransmitters, systems neuroscience, behavior, and self-report).

Taken together, the review highlights an urgent and clear need to develop harmonized ways of assessing agency. Future research needs to either develop a gold-standard comprehensive agency task, or to use a battery of comparable tasks when exploring agency within and across conditions. Agreed-upon measure(s) of agency will undeniably provide a stronger foundation for identifying similarities and differences across disorders. In our view, an ideal task (or battery of tasks) would involve: (i) a cognitive task contrasting an active condition with a passive condition to facilitate quantification of over- and under-attribution of agency, (ii) in predictable and uncertain conditions to compare baseline best-case-scenario performance and simulate the complexities of everyday inferences, (iii) incorporating agency an implicit measure (the best likely candidate being a temporal perception measure, though alternatives exist, e.g., neural measures), (iv) as well as explicit questions about the attribution of agency to task actions and events. This should be supported by generalised questions about agentive experience in daily life, such as the Sense of Agency Scale questionnaire (Tapal et al., 2017). Together, a measure with these features would create a rich picture to uncover the differences in agency processes and experience between groups. Further, future research investigating different dimensions of agency, across clinical stages, and using longitudinal data will clarify if altered agency is a useful transdiagnostic dimension that reflects of illness progression, and may contribute to identifying a set of informative psychiatric traits in the healthy spectrum.

## Data availability statement

The original contributions presented in the study are included in the article/Supplementary material, further inquiries can be directed to the corresponding author.

## Author contributions

DT: Conceptualization, Data curation, Investigation, Methodology, Project administration, Validation, Writing – original draft. OC: Conceptualization, Methodology, Project administration, Supervision, Validation, Writing – review & editing. D-RM: Data curation, Investigation, Writing – review & editing. KP: Conceptualization, Data curation, Investigation, Methodology, Project administration, Supervision, Validation, Visualization, Writing – review & editing.
