# Supplementary material for: Agency in schizophrenia and autism: a systematic review
Source: Front Psychol. 2023 Dec 21;14:1280622. doi: 10.3389/fpsyg.2023.1280622 (PMC10768057; doi:10.3389/fpsyg.2023.1280622)
Supplement: Supplementary file 1 [file Table_1.DOCX]

Supplementary Material

# Search Strategy

## PsycINFO & Embase & Medline

((Schizophren* OR Schizophrenia spectrum* OR Psychotic OR Psychosis OR Delusion* OR Hallucination* OR Paranio* OR Formal thought disorder* OR First rank symptom*) AND (Sense of agency OR Feeling* of agency OR Judg* of agency OR Projected hand illusion OR Action monitoring OR Intentional binding OR Temporal binding))

((Autis* OR ASD OR Asperger* OR Pervasive development*) AND (Sense of agency OR Feeling* of agency OR Judg* of agency OR Projected hand illusion OR Action monitoring OR Intentional binding OR Temporal binding))

((Autis* OR ASD OR Asperger* OR Pervasive development*) AND (Schizophren* OR Schizophrenia spectrum* OR Psychotic OR Psychosis OR Delusion* OR Hallucination* OR Paranio* OR Formal thought disorder* OR First rank symptom*) AND (Sense of agency OR Feeling* of agency OR Judg* of agency OR Projected hand illusion OR Action monitoring OR Intentional binding OR Temporal binding))

## Web of Science

((Schizophren* OR Schizophrenia spectrum* OR Psychotic OR Psychosis OR Delusion* OR Hallucination* OR Paranio* OR “Formal thought disorder*” OR “First rank symptom*”) AND (“Sense of agency” OR “Feeling* of agency” OR “Judg* of agency” OR “Projected hand illusion” OR “Action monitoring” OR “Intentional binding” OR “Temporal binding”))

((Autis* OR ASD OR Asperger* OR “Pervasive development*”) AND (“Sense of agency” OR “Feeling* of agency” OR “Judg* of agency” OR “Projected hand illusion” OR “Action monitoring” OR “Intentional binding” OR “Temporal binding”))

((Autis* OR ASD OR Asperger* OR “Pervasive development*”) AND (Schizophren* OR Schizophrenia spectrum* OR Psychotic OR Psychosis OR Delusion* OR Hallucination* OR Paranio* OR “Formal thought disorder*” OR “First rank symptom*”) AND (“Sense of agency” OR “Feeling* of agency” OR “Judg* of agency” OR “Projected hand illusion” OR “Action monitoring” OR “Intentional binding” OR “Temporal binding”))

## Pub Med

((Schizophren*) OR (Schizophrenia spectrum*)) OR (Psychotic)) OR (Psychosis)) OR (Delusion*)) OR (Hallucination*)) OR (Paranoi*)) OR (Formal thought disorder*)) OR (First rank symptom)) AND ((((((("Action monitoring") OR ("Intentional binding")) OR ("Projected hand illusion")) OR ("Temporal binding")) OR ("Sense of agency")) OR (feeling* agency)) OR (judg* agency))

((Autis*) OR (ASD)) OR (Asperger*)) OR (Pervasive development*)) AND ((((((("Action monitoring") OR ("Intentional binding")) OR ("Projected hand illusion")) OR ("Temporal binding")) OR ("Sense of agency")) OR (feeling* agency)) OR (judg* agency))

(((Schizophren*) OR (Schizophrenia spectrum*)) OR (Psychotic)) OR (Psychosis)) OR (Delusion*)) OR (Hallucination*)) OR (Paranoi*)) OR (Formal thought disorder*)) OR (First rank symptom)) AND ((((Autis*) OR (ASD)) OR (Asperger*)) OR (Pervasive development*))) AND ((((((("Action monitoring") OR ("Intentional binding")) OR ("Projected hand illusion")) OR ("Temporal binding")) OR ("Sense of agency")) OR (feeling* agency)) OR (judg* agency))Supplementary Figures and Tables

## Supplementary Tables

### Supplementary Table 1 Eligibility Criteria and Study Selection

|  | **Inclusion Criteria (Yes/No)** | **Exclusion Criteria** |
| --- | --- | --- |
| **Language** | - Is the article in English? |  |
| **Document type** | - Is the research article found in a journal? - Is it peer reviewed? |  |
| **Publication date** | - Is it published after 1994 (DSM-IV onwards)? - Exception: For papers on Schizotypy in healthy population, publication from DSM-III will be included. | DSM-III and before (with exception of papers measuring schizotypy in healthy population) |
| **Data Type** | - Does the study use primary and quantitative data? - Does the study, if intervention included, assess outcome measure at baseline? | - Reviews - Meta-analyses - Self-report data - Case studies - Intervention studies that did not assess outcome measure at baseline - Books, if only include the above |
| **Participants** | - Are the participants human? - Are the healthy controls neurotypical and indicate absence of psychiatric diagnoses? - Do the participants in clinical groups meet diagnostic threshold for either Autism Spectrum Disorder or Schizophrenia Spectrum Disorder? - Schizophrenia diagnoses may include Schizophrenia disorder, Schizoaffective disorder, Brief Psychotic Disorder, Schizophreniform Disorder - Autism diagnoses may include: Autism Spectrum Disorder (ASD), Asperger’s Syndrome, Pervasive Developmental Disorder (PDD), PDD-NOS. - Do the participants in clinical groups meet sub-threshold standards for Autism or Schizophrenia? If so, indicate for future consideration. - Autism Quotient has been commonly used as measure of sub-threshold Autism - Schizotypy to be labelled for future review - Do the participants have co-morbid psychiatric illnesses? If so, include and indicate. - Were the diagnoses informed by DSM-IV onwards (i.e., DSM-IV, DSM-IV-TR, DSM-V) or ICD-10 onwards (i.e., ICD-10, ICD-11)? - Were the clinical diagnoses provided by a psychiatrist/psychologist/ medical doctor/ other mental health clinician trained in mental health utilising structured clinical interview (e.g., SCID, ADOS, Asperger Syndrome Diagnostic Interview, ADI-R)? - Were the clinical diagnoses identified through medical file review? - Were the diagnoses identified in other ways? If so, indicate. | - Animal - Diagnosis informed by criteria outlined in DSM-III and earlier - Diagnostic groups like Ultra-high Risk and prodromal risk for psychosis - Diagnosis identified by patient’s self-report and not by a professional |
| **Study design** | - Does the study use a case-controlled trial design? - Does the study include a comparison between healthy controls and clinical group (i.e., between ASD and healthy control, between SZD and healthy control)? - Does the study include a comparison between clinical groups (i.e., between ASD and SZD) - Does the study compare outcome measures that are related to agency? - If the paper the studying schizotypal traits or ASD traits within healthy population, comparison with a control group is not necessary. - If the paper directly compares ASD and Schizophrenia, a healthy group is not necessary. | Studies with no comparisons between control and clinical group (with exception of comparisons between ASD and SZD) |
| **Outcome type** | - Does the study use cognitive/ social/ behavioural paradigms to measure agency (judgement/ sense/ feeling of agency)? - Does the study use implicit (e.g., intentional binding) or explicit measures (judgement of agency, likert scale)? | Studies using biological or neurological correlates or neuroimaging techniques only |
| **Outcome measure** | - Does the study use behavioural/ performance/ task-based measures? - If measuring judgement of agency, does the study use a self-report element to the task---as in “do you experience agency over your actions?” - Does the study include an active condition/ component for agency measures (i.e., self-initiated actions performed by participants in action monitoring task/ intentional binding task)? - Does the study have at least one condition that is self-focused? | Studies using neuroimaging only (include if behavioural component present)  Studies using genetic investigation  Studies that only explore body-part ownership than agency (E.g., rubber hand illusion) |
